# pH-Responsive Mercaptoundecanoic Acid Functionalized Gold Nanoparticles and Applications in Catalysis

**DOI:** 10.3390/nano8050339

**Published:** 2018-05-17

**Authors:** Siyam M. Ansar, Saptarshi Chakraborty, Christopher L. Kitchens

**Affiliations:** 1 Department of Chemical and Biomolecular Engineering, Clemson University, Clemson, SC 29634, USA; mmohame@g.clemson.edu (S.M.A.); saptarc@g.clemson.edu (S.C.); 2 Institute of Environmental Toxicology (CU-ENTOX), Clemson University, 509 Westinghouse Road, Pendleton, SC 29670, USA

**Keywords:** gold nanoparticles, MUA, aggregation and redispersion, phase transfer, reusable catalyst

## Abstract

Mercaptoundecanoic acid (MUA) functionalized gold nanoparticles (AuNP-MUA) were synthesized and demonstrated to possess pH-triggered aggregation and re-dispersion, as well as the capability of phase transfer between aqueous and organic phases in response to changes in pH. The pH of aggregation for AuNP-MUA is consistent with the *pK_a_* of MUA (pH ~4) in solution, while AuNP-MUA phase transition between aqueous and organic phases occurs at pH ~9. The ion pair formation between the amine group in octadecylamine (ODA), the carboxylate group in MUA, and the hydrophobic alkyl chain of ODA facilitates the phase transfer of AuNP-MUA into an organic medium. The AuNP-MUA were investigated as a reusable catalyst in the catalytic reduction of 4-nitrophenol by borohydride—a model reaction for AuNPs. It was determined that 100% MUA surface coverage completely inhibits the catalytic activity of AuNPs. Decreasing the surface coverage was shown to increase catalytic activity, but this decrease also leads to decreased colloidal stability, recoverability, and reusability in subsequent reactions. At 60% MUA surface coverage, colloidal stability and catalytic activity were achieved, but the surface coverage was insufficient to enable redispersion following pH-induced recovery. A balance between AuNP colloidal stability, recoverability, and catalytic activity with reusability was achieved at 90% MUA surface coverage. The AuNP-MUA catalyst can also be recovered at different pH ranges depending on the recovery method employed. At pH ~4, protonation of the MUA results in reduced surface charge and aggregation. At pH ~9, ODA will form an ion-pair with the MUA and induce phase transfer into an immiscible organic phase. Both the pH-triggered aggregation/re-dispersion and aqueous/organic phase transfer methods were employed for catalyst recovery and reuse in subsequent reactions. The ability to recover and reuse the AuNP-MUA catalyst by two different methods and different pH regimes is significant, based on the fact that nanoparticle-catalyzed reactions may occur under different pH conditions.

## 1. Introduction

Gold nanoparticles (AuNPs) have attracted significant interest due to their optical, electronic, and chemical properties, which have demonstrated potential applicability in a variety of fields, including chemical catalysis [1,2,3]. While AuNPs possess inherent properties, surface functionalization with a variety of ligands affords the enhancement of existing properties or the introduction of additional capabilities that make the functionalized AuNPs suitable for novel applications. For example, AuNPs functionalized with external stimuli-responsive molecules possess chemical or physical properties that are triggered by light, pH, temperature, ions, or other stimuli, which have a significant potential for applications in reusable catalysis, sensory devises, biomedical applications, etc. [4,5,6,7,8].

AuNP functionalized with pH-responsive groups, such as carboxylic acids, sulfonates, and amines, have been synthesized and possess pH-responsive behaviors in solution [4,9,10,11]. For example, 11-mercaptoundecanoic acid (MUA) is a pH-responsive ligand that binds strongly to AuNPs through the thiol group and effectively disperses nanoparticles in water at neutral and basic pH levels. MUA-stabilized AuNPs (AuNP-MUA) have been synthesized, and their colloidal behavior has been studied as a function of pH, ionic strength, and amine-induced AuNP-MUA aggregation in water [12,13,14]. Su et al. synthesized the MUA-functionalized 13 nm AuNPs via ligand exchange between citrate and MUA, and studied the colloidal stability and phase behavior [15]. They believed that the aggregation of AuNP-MUA at pH 3 is governed by hydrogen-bonding forces between the surface adsorbed MUA molecules. At pH 11, the AuNP-MUA are colloidally stable in solution but form three-dimensional close-packed aggregates on TEM grids, due to decreased electrostatic repulsion interactions between deprotonated MUA and counter-ions (Na^+^) during the sample drying process. Recently, Pillai et al. studied the nanoparticle size effect on the precipitation pH (pH^prec^) for AuNPs functionalized with a mixed monolayer of MUA and *N,N,N*-trimethyl (11-mercaptoundecyl) ammonium ion [12]. They found that the pH^prec^ increased from pH = 5.3 to pH = 7.3 when increasing the nanoparticle size from 4.2 to 11.5 nm. Laaksonen et al. studied the stability of 2.3 nm AuNP-MUA at a set pH, using the hydroxide as base and varying the size of counter-ions, and showed that AuNP-MUA aggregation occurred at 70–90 mM for Na^+^, and at greater than 1 M for the quaternary ammonium cation [13]. The steric hindrance caused by the quaternary ammonium adsorbed in the Stern layer stabilized the AuNP-MUA against aggregation. Recently, Wang et al. studied the stability of 4–6 nm AuNP-MUA to different monovalent cations that have different propensities for bridging interactions, as well as for concomitant AuNP-MUA aggregation [14]. The authors showed that the order of salt concentrations needed for AuNP-MUA aggregation is CsCl >> KCl > LiCl > NaCl > RbCl, which does not correlate with the size of the hydrated cations.

Though AuNP-MUA aggregation and redispersion in water has been explored before, the pH-triggered AuNP-MUA phase transfer between the water and organic phases (without aggregation) and reuse in catalysis has been not reported. While understood phenomenologically, there is a fundamental tradeoff between colloidal stability and catalytic activity, which is driven by nanoparticle ligand surface passivation. This understanding is integral to the design of colloidal catalysts with sufficient activity and the ability to be recovered and reused in subsequent reactions. Our approach is to use stimuli-responsive surface functional groups for the catalyst recovery and reuse; however, the challenge is to balance the degree of surface coverage where higher passivation promotes colloidal stability and preservation of the nanoparticle catalyst, but also inhibits activity. Recently, we studied the catalytic activity of thiolated polyethylene glycol (PEG) ligands with varying chain lengths and surface coverage for the catalytic 4-nitrophenol reduction reaction [16]. Our results demonstrated an inverse correlation between catalytic activity and PEG surface coverage on the AuNPs.

In this work, we perform an in-depth study of pH-triggered AuNP-MUA aggregation and redispersion, as well as AuNP phase transfer between water and organic phases. This phase behavior is then coupled with application as a recoverable and reusable colloidal catalyst. Our results show that MUA provides pH-responsive dispersibility and phase transferability between aqueous and organic media, with the addition of a pH-responsive phase transfer facilitator. The activity of AuNP-MUA in the catalyzed reduction of 4-nitrophenol (4-NP) to 4-aminophenol (4-AP) by sodium borohydride (NaBH_4_) was explored. AuNP-MUA are catalytically active towards the reduction of 4-NP to 4-AP at lower MUA surface coverage; however, low surface coverage also results in decreased recovery and reusability. We have explored this tradeoff for AuNP-MUA and demonstrated the ability to achieve pH-triggered AuNP-MUA phase transfer between the water and organic phase (without aggregation) and reuse, without loss in catalytic activity.

## 2. Materials and Methods

### 2.1. Chemicals and Equipment

Toluene was purchased from Alfa Aesar (Tewksbury, MA, USA). All other chemicals were acquired from Sigma Aldrich (St. Louis, MO, USA). No further purification was conducted on the chemicals. Ultra-pure Milli-Q water (resistivity 18.2 MΩ.cm) was used for all synthesis and reactions. A pH meter (sympHony SB90M5, VWR International, Radnor, PA, USA) was used to measure pH. UV-VIS spectra were acquired on a UV-VIS Spectrometer (Varian Cary 50, Agilent Technologies, Santa Clara, CA, USA).

### 2.2. Mercaptoundecanoic Acid (MUA) Functionalized AuNP Synthesis

Borohydride reduction was employed to synthesize citrate-stabilized 5 nm diameter AuNPs. The citrate reduction method was used for synthesizing 13 and 45 nm diameter AuNPs [17,18,19]. A mixture of 0.5 mM HAuCl_4_ (50 mL) and 0.5 mM trisodium citrate was made in a conical flask. A solution of 0.1 M sodium borohydride (1.5 mL of ice-cold, freshly prepared) was subsequently added dropwise under constant stirring. Stirring was continued for an additional hour. For 13-nm AuNPs, 150 mL of 1 mM HAuCl_4_ aqueous solution was heated while gently stirring. When the solution begins to boil, 5.0 mL of 120-mM citrate in H_2_O was added, and the resulting solution was stirred at 400 rpm for 15 min as the color of the solution changed from colorless to red. Two-step nanoparticle seeded growth method was used to synthesize the citrate-capped 45-nm AuNPs [19]. In brief, 10 mL of as-synthesized 13 AuNP was added to 150 mL of boiling solution containing 0.6 mM HAuCl_4,_ followed by addition of 1% *w*/*w* aqueous trisodium citrate (21.7 µmol, 1.3 mL). The mixture was heated for 30 min under vigorous stirring.

Citrate-stabilized AuNPs were ligand exchanged with thiolated MUA to generate AuNP-MUA. A total of 1.3 mM MUA (30 mL, dilute NaOH) and 10 mL of as-synthesized citrate-AuNP were incubated for 24 h. AuNP-MUA was washed by repeated centrifugal precipitation and re-dispersion three times with H_2_O, to remove excess MUA.

### 2.3. Thermogravimetric (TGA) Analysis 

The quantity of MUA grafted to AuNP was measured through TGA (SDT Q600, TA Instruments, New Castle, DE, USA). On a TGA pan (alumina), 50 mL purified AuNP-MUA was reduced down to 60 µL by repeated centrifugation (14,500 rpm, 1 h) and deposited. Water was removed initially by holding the TGA temperature at 100 °C for 15 min. A temperature ramp of 10 °C/min was applied till a final temperature of 600 °C was achieved and the temperature was held for 15 min (N_2_ purge, 20 mL/min).

### 2.4. 4-Nitrophenol Reduction Catalysis

Time-resolved UV-VIS spectra was acquired in a 4 mL quartz cell (Varian Cary 50 spectrophotometer). AuNP-MUA (1 mL), H_2_O (0.9 mL), and 0.2 mM 4-NP (1 mL) were mixed in the quartz cell. Change in intensity of 4-NP peak at 400 nm as function of time (individual spectra were acquired every 0.2 min) was used to track reaction progress.

### 2.5. Transmission Electron Microscopy (TEM) Analysis

Hitachi 9500 (300 kV, Hitachi, Schaumburg, IL, USA) was used to acquire high-resolution TEM images of AuNPs, and ImageJ size analysis was conducted on the images. Then 10 µL of AuNP was drop cast on a 300 mesh Cu grids (Formvar coated) and allowed to dry. TEM grids were subsequently stored in a desiccator for complete removal of solvent.

### 2.6. Dynamic Light Scattering (DLS) Measurements

DLS measurements were made on five-times-diluted as-prepared AuNPs at 25 °C (Malvern instrument Zeta sizer Nano series, Westborough, MA, USA). The solutions were adjusted to the desired pH with either 0.1 M HCl or 0.1 M NaOH solutions, and their hydrodynamic diameters (number averaged) and ζ potentials were measured.

## 3. Results and Discussion

**Figure 1:**
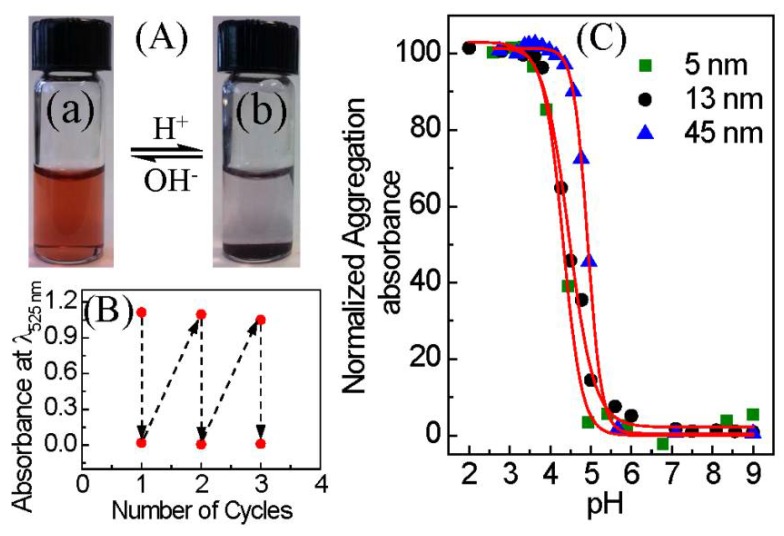
(**A**) Photographs showing the reversibility of 13-nm mercaptoundecanoic acid functionalized gold nanoparticles (AuNP-MUA) clustering/re-dispersion by changing the pH of the medium. The left vial (a) contains well-dispersed AuNP-MUA at a basic pH, and the right vial (b) contains aggregated and settled AuNP-MUA at an acidic pH; (**B**) Plot showing the pH-triggered reversibility of aggregation and re-dispersion monitored by the localized surface plasmon resonance (LSPR) peak intensity at 525 nm for 13-nm AuNP-MUA; and (**C**) normalized UV-VIS absorbance peak ratio of aggregated and unaggregated AuNP-MUA as a function of aqueous phase pH. The absorbance for un-aggregated 5, 13, and 45 nm diameter AuNPs were measured at wavelengths of 522, 525, and 551 nm, respectively, and the absorbance for aggregated 5, 13, and 45 AuNPs were measured at wavelengths of 562, 595, and 725 nm, respectively.

Citrate-capped AuNPs with three different sizes (5, 13, and 45 nm) were first synthesized by the borohydride and citrate reduction methods [17,18,19]. Transmission electron microscopy (TEM) shows that the average sizes of as-synthesized AuNPs are 4.6 ± 1.9, 13.4 ± 1.1, and 45.9 ± 5.9 nm in diameter (Supplementary Materials, Figure S1). MUA-stabilized AuNPs were prepared by a ligand exchange reaction between citrate-stabilized AuNPs and the MUA in dilute KOH. Dynamic light scattering (DLS) data for AuNPs before and after MUA functionalization demonstrate the colloidal stability of nanoparticles in a dilute KOH solution (See Supplementary Materials). The UV-VIS spectra of AuNPs-MUA exhibit a characteristic localized surface plasmon resonance (LSPR) absorption at 510–560 nm, confirming the stability of the basic medium (Supplementary Materials, Figure S2). MUA strongly binds with AuNPs through covalent bonding of the thiol to the gold surface, yielding a pH-responsive –COOH group at the distal end. Figure 1A shows the pH-responsiveness of AuNP-MUA, undergoing reversible aggregation/precipitation and re-dispersion at an acidic and basic pH, respectively. Wine-red color solution (left vial) indicates well-dispersed nanoparticles in basic medium. In acidic medium (dilute HCl is used to adjust pH with mild stirring), the nanoparticles aggregate immediately and precipitate over an hour of incubation, leading to the complete settling of nanoparticles (right vial). The strong LSPR peak is used to monitor the aggregation and redispersion of AuNPs by UV-VIS spectroscopy (Supplementary Materials, Figure S3). The complete disappearance of the peak at an acidic pH indicates that aggregated nanoparticles completely settled out. Complete re-dispersion of the precipitated AuNPs (dilute KOH is used for pH adjustment with mild stirring) is evident by the complete recovery of the LSPR peak at 526 nm and absorbance of approximately 1.1, accounting for dilution. Figure 1B shows the reversibility of the AuNP-MUA (13 nm particles) aggregation/re-dispersion process for several cycles. Other sizes of AuNP-MUA (5 and 45 nm particles) exhibit the same reversible aggregation and re-dispersion (data not shown). 

The pH of AuNP-MUA aggregation was determined from a UV-VIS absorbance titration curve (Figure 1C) obtained by monitoring the peak maximum absorbance for aggregated and un-aggregated peaks at different pH values (Supplementary Materials, Figure S4). The LSPR peak at 510–560 nm for well-dispersed AuNPs peak shifts to a higher wavelength, and decreases in intensity as the AuNPs aggregate and settle [20,21]. The pH of AuNP-MUA aggregation (pH_agg_) is determined from the inflection point of a sigmoidal fit of the absorbance, yielding pH values of 4.3, 4.5, and 4.9 for 5 nm, 13 nm, and 45 nm particles, respectively. Pillai et al. also observed similar trend for a MUA and *N*,*N*,*N*-trimethyl (11-mercaptoundecyl) ammonium ion mixed monolayer functionalized 4.2–11.5 nm AuNPs [12]. Also, Wang et al. reported that the *pK_a_* value of MUA bound to AuNPs increases with increasing nanoparticle size from 4.1 to 7.2 nm [22]. Therefore, it is clear that as the particle size increases (nanoparticle curvature reduces), the deprotonation of the –COOH group on the nanoparticle surface is inhibited, due to the strong electrostatic repulsions between the carboxylate ions. In other words, at a given pH value, the fraction of –COO^−^ (compared to –COOH) on the AuNP surface increases as the nanoparticle size decreases, which corresponds to the pH_agg_ increase with nanoparticle size. Furthermore, it should be noted that the wavelength and intensity of the LSPR peak of plasmonic nanoparticles is very sensitive to the dielectric properties of the local environment of the nanoparticles and the interparticle interaction (particle spacing) of nanoparticles [23,24]. Thus, to delineate these effects, measurement of the apparent diffusion coefficient and hydrodynamic diameter by DLS can complement the UV-VIS as an in situ measurement of nanoparticle aggregation as a function of pH.

**Figure 2:**
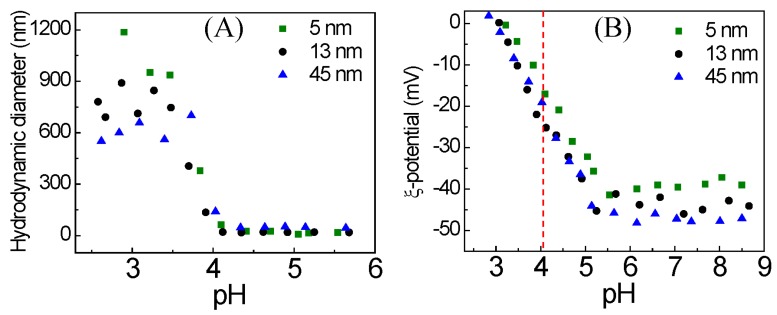
(**A**) Hydrodynamic diameter of the AuNP-MUA as a function of pH; and (**B**) ζ-potential of the AuNP-MUA as a function of pH. The red line indicates the onset of AuNP-MUA aggregation based on the hydrodynamic diameter data from figure A.

The onset pH of AuNP-MUA aggregation determined by the DLS titration curve, as evidenced by increasing hydrodynamic diameter (Figure 2A), commences at a pH of about 4.1 for all the three sizes of AuNPs-MUA. Our DLS results for pH at onset of AuNP-MUA aggregation are consistent (within the same pH units) with the data obtained from the UV-VIS titration method (Figure 1C). As can be seen in Figure 2B, at higher pH (>5), the ζ-potential is highly negative due to the deprotonated carboxylate group of MUA, which provides electrostatic repulsion between AuNP-MUA and thus colloidal stability. The magnitude of the ζ-potential is commonly used as the measure of colloidal stability, neglecting steric contributions [25,26,27]. Once the pH decreases below 5, the ζ-potential dramatically decreases, due to the protonation of the carboxylate groups over the pH range from 5 to 3. As a result, the electrostatic repulsion between nanoparticles decreases, eventually leading to nanoparticle aggregation. The decreasing magnitude of the AuNP-MUA ζ-potential as a function of pH indicates that the onset of aggregation, with an increased hydrodynamic diameter at pH 4.1, occurs at a ζ-potential of ~ −20 mV for all the sizes of particles. Thus, the ζ-potential data indicate that the AuNP-MUA aggregate with an approximately 50% reduction of surface charge. Indeed, the ζ-potential is not quite equivalent to the surface charge on AuNPs; also, the ζ-potential of AuNP-MUA is dependent not only on the *pK_a_* of surface-adsorbed MUA, but also on the MUA packing density and the surrounding environment. 

Therefore, the ζ-potential and hydrodynamic data also confirm that the pH_agg_ of AuNP-MUA is ~4.1, which is comparable with *pK_a_* ≈ 4.8 for MUA in solution [28]. However, the pH_agg_ for AuNP-MUA is about two pH unit smaller than the reported *pK_a_* value for MUA adsorbed on AuNPs [22,29]. Recently, Charron et al. reported the *pK_a_* value of MUA adsorbed onto 5 nm AuNP by titrating with NaOH (acid-base titration method) [29]. They reported the *pK_a_* value of MUA adsorbed on AuNP is around 7, which suggests a *pK_a_* about two pH units higher than that of the unbound MUA. Wang et al. studied the dissociation behavior of AuNP-tethered MUA as a function of pH, using an acid-base (or potentiometric) titration method [22]. They also observed similar phenomena for the *pK_a_* of MUA bound to 7.2 nm AuNPs increased to ~8.3, which is significantly higher than that of MUA in solution (*pK_a_* ≈ 4.8) [22].

**Figure 3:**
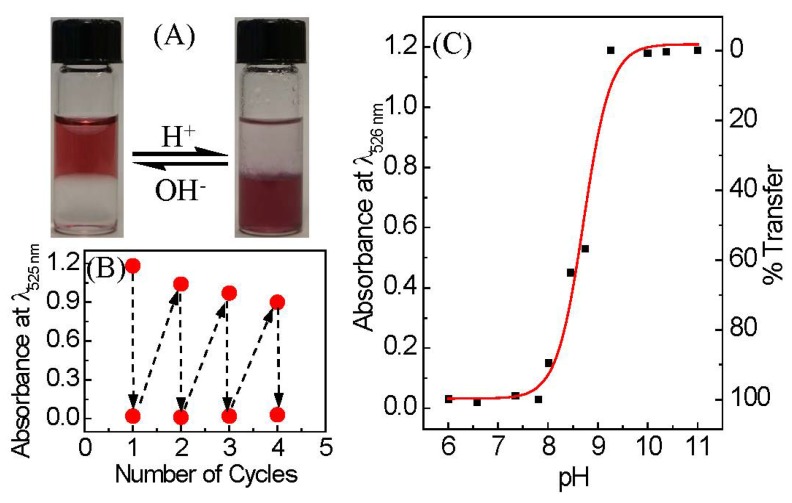
(**A**) Photographs of the pH-triggered reversible phase transfer of 13-nm AuNP-MUA between water and CHCl_3_ layers, by switching the pH. The left side vial contains well-dispersed AuNP-MUA in the aqueous phase (top layer) at basic pH, and the right side vial contains AuNP-MUA transferred into the CHCl_3_ phase (bottom) layer after adding HCl and vigorous shaking; (**B**) Plot showing pH-triggered reversible phase transfer of 13 nm AuNP-MUA between the water and organic phase, by monitoring the AuNP-MUA LSPR peak intensity at 525 nm wavelength in aqueous phase; and (**C**) absorbance of AuNP-MUA in aqueous phase at 525 nm (left scale) versus the pH and percentage transfer of AuNP-MUA from an aqueous to a CHCl_3_ layer as a function of pH. The percentage of transfer was calculated by taking the absorbance of the AuNP-MUA (in aqueous medium) at pH 11.0 as 0%. The red color solid curve represents sigmoidal fitting of the experimental data.

Direct transfer of nanoparticles from aqueous to organic phases is frequently employed in nanoparticle synthesis and purification applications [30,31]. In some colloidal nanoparticle catalytic applications, phase transfer of nanoparticles between two immiscible liquids is extremely advantageous for the recycling and reuse of catalysts, due to the avoidance of irreversible nanoparticle aggregation. To date, many methods used to modulate nanoparticle phase transfer have been developed, such as host–guest interactions [32,33], electrostatic interactions [34,35,36], covalent modifications [37], and ligand exchanges [38,39,40,41,42]. It must be mentioned that with many of these methods, reversible phase transfer is not achieved; however, for certain applications, irreversible phase transfer is preferred. Here we demonstrate reversible pH-triggered phase transfer of 13 nm AuNP-MUA between the aqueous and organic phases. AuNP-MUA in an aqueous phase are transferred into a CHCl_3_ layer, by reducing the aqueous layer pH from 11.0 to 8.0 with 0.1 M HCl and vigorous mixing for 2 min (Figure 3A). The phase transfer occurs only in the presence of octadecylamine (ODA), which acts as the phase transferring agent when the ODA is protonated (charged) at pH below the *pK_a_* and is deprotonated at pH above the *pK_a_*. In short, a pH of 1.5 mL of AuNP-MUA (pH = 11) was adjusted to 8.0 with HCl, and subsequently vigorously agitated with 1.5 mL of chloroform containing ~1 mg ODA for 2 min. The necessity of ODA as a phase-transferring agent is demonstrated with a control experiment where AuNP-MUA aggregates on the vial surface and water-chloroform interface when acidic pH is employed without using ODA. (Supplementary Materials, Figure S5). Phase transfer between aqueous and organic phases is reversible for at least four cycles (Figure 3B), as indicated by monitoring the LSPR peak of AuNP-MUA in the aqueous layer (Supplementary Materials, Figure S6). The pH for the phase transition (pH_trans_) of 8.7 was determined from the inflection point of a sigmoidal fit of the percentage of AuNP-MUA transferred from aqueous to organic layers as a function of pH, determined from the LSPR peak absorbance in the aqueous layer (Figure 3C and Supplementary Materials, Figure S7). The transfer from aqueous to organic phase occurs when the pH of the aqueous layer is below the *pK_a_* of the amine headgroup in ODA (~10.6) and above the *pK_a_* of MUA (~4.5) (Figure 3C). The phase transfer into the organic phase is due to the ion-pair formation between a negatively-charged carboxylate group (above pH ~4) and the positively-charged amine group of ODA (below pH 10.6). The long hydrophobic alkyl chain of ODA makes the AuNP-MUA more hydrophobic via its ion-pair formation. AuNP-MUA (1.8 nm diameter) phase transfer to organic phase by binding to highly-hydrophobic cationic molecules, such as tetraoctylammonium, has been reported previously [43]. Recently, Yuan et al. demonstrated a phase transfer cycle (aqueous → organic → aqueous) where glutathione functionalized Ag, Au, Cu, and Pt nanoparticles (<2 nm diameter) have been transferred into toluene or hexane via electrostatic interaction between negatively charged carboxylate groups on metal nanoparticles and positively charged cetyltrimethylammonium (CTA^+^, hydrophobic) [34]. The removal of CTA^+^ from the nanoparticle by forming a hydrophobic salt between tetramethylammonium decanoate and CTA^+^ restores the negative charge on the nanoparticle surface, and returns the nanoparticles back to the aqueous phase. In this work, we have shown that the AuNP-MUA can easily and reversibly separate from an aqueous phase by either aggregation or phase separation methods.

**Figure 4:**
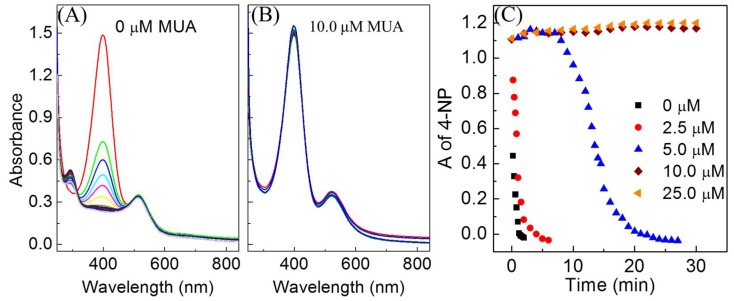
Catalytic activity of AuNP-MUA as function of MUA packing density on AuNPs. (**A**) Time-resolved UV-VIS spectra of 4-nitrophenol (4-NP) reduction reaction catalyzed by AuNPs functionalized with 0 µM MUA; (**B**) Time-resolved UV-VIS spectra of 4-nitrophenol reduction reaction catalyzed by AuNPs and functionalized with 10 µM MUA; and (**C**) The progress of the reaction tracked by the change in 4-NP absorbance peak at 400 nm over the time.

The ability to reversibly induce AuNP-MUA separation and re-dispersion is only half of the equation for colloidal catalysis; it must also possess catalytic activity. The catalytic activity of 13 nm AuNP-MUA was tested with the 4-nitrophenol (4-NP) reduction by borohydride, which is a common model reaction for ligand-modified AuNPs [44,45,46,47]. Figure 4A and Supplementary Materials Figure S8 show the time-resolved UV-VIS spectra of a 4-NP reduction reaction catalyzed by AuNP, as a function of MUA surface coverage. The MUA surface coverage on the AuNPs was controlled by stoichiometry—mixing different concentrations of MUA with AuNPs during the ligand exchange process. MUA surface coverage on AuNP for 1 mM MUA with the AuNPs sample was determined by thermogravimetric analysis (TGA) (Supplementary Materials, Figure S9). The percentage weight loss of MUA adsorbed onto AuNPs is 3.7%, corresponding to the MUA monolayer packing density on AuNPs of 4.56 molecules/nm^2^ (See Supplementary Materials), which is comparable to previously reported MUA packing density on AuNPs (5.70 molecules/nm^2^) [48]. MUA surface coverage at different concentrations of MUA in an AuNP ligand exchange was determined using the 4.56 molecules/nm^2^ monolayer packing density. The estimated surface coverages on AuNPs are 0%, 30%, 60%, 100%, and 100% for 0, 2.5, 5.0, 10.0, and 25.0 µM MUA in the ligand exchange reaction, respectively (See Supplementary Materials). Figure 4B shows the kinetics of the reaction monitored in situ using time-resolved UV-VIS spectroscopy, via changes in intensity of the 4-NP peak at 400 nm [44,45,46,47]. No reaction was observed for the 100% MUA surface coverage, which is expected due to complete thiol binding to all catalytic sites. At surface coverages below 100%, the AuNP-MUA are active in catalyzing the 4-NP reduction. Furthermore, an induction time was observed for 60% of the MUA surface coverage sample (Figure 4B). Induction time is generally observed in ligand stabilized colloidal catalysts, and occurs due to mass transfer resistance offered by the ligand [46,49] or slow surface restructure due to adsorbed reactants [50,51,52]. A similar phenomenon has been observed in our prior work for the catalytic activity of thiolated PEG functionalized AuNPs, where increased induction time coincided with increased surface coverage [16].

**Figure 5:**
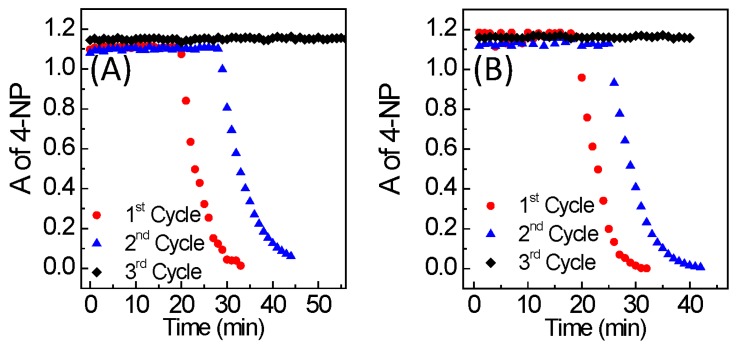
Recovery and reuse of AuNP-MUA with 90% surface coverage in catalysis by using (**A**) pH-triggered aggregation/redispersion method and (**B**) pH-triggered phase transformation method.

While catalytic activity was observed with 0%, 30%, and 60% MUA surface coverage, the decreased surface coverage did not provide sufficient colloidal stability. As such, the AuNPs could not be recovered and re-dispersed by aggregation/re-dispersion or phase transfer methods following the catalytic reaction. However, the pH-triggered reversible phase transfer and aggregation/re-dispersion of AuNP-MUA was achieved with 60% surface coverage in the absence of the reaction (Supplementary Materials, Figure S10). In order to enhance the colloidal stability, the surface coverage was increased to 90% by increasing the MUA concentration to 7.5 µM. Ninety percent of surface coverage on AuNP is catalytically active, despite longer induction times on the order of 20 min (Figure 5). More importantly, 90% of surface coverage AuNP-MUA was successfully recovered after the first reaction cycle, and reused in a second catalytic cycle by both aggregation/re-dispersion and phase transfer methods. In the second cycle, the catalytic induction times were increased, but 100% 4-nitrophenol conversation was maintained. The rate constant is obtained by fitting the data from Figure 5 to pseudo-first-order reaction kinetics with respect to 4-nitrophenol, and the rate constant is indicative of catalytic activity (Supplementary Materials, Figure S11). The reaction rate constants for the catalysts recovered by aggregation/re-dispersion method are 0.29 ± 0.04 and 0.20 ± 0.06 min^−1^ for the first and second cycles, respectively, and the rate constants for the phase transfer method are 0.31 ± 0.03 and 0.23 ± 0.05 min^−1^ for the first and second cycles, respectively. Unfortunately, the catalytic activity was lost for the third catalytic cycle, due to the irreversible aggregation of AuNP-MUA during the recovery processes.

## 4. Conclusions

We have demonstratedthat pH controls the dispersion of MUA-functionalized AuNPs where reversible aggregation and redispersion in an aqueous phase is achieved around pH 4.1 or the *pK_a_* of MUA. Furthermore, reversible phase transfer between aqueous and organic phases (toluene or CHCl_3_) can be achieved with the use of an amine-containing phase transfer agent (ODA) at pH 8.7 or the *pK_a_* of the amine, where an ion pair formation induces phase transfer to chloroform. The catalytic activity of AuNPs functionalized with different surface coverages of MUA were studied. Complete inhibition of catalytic activity was observed at 100% surface coverage of MUA. AuNPs with 60% and less MUA surface coverage were colloidally stable and catalytically active, but possessed poor recoverability and reusability following the reactions. In this system, there is a tradeoff between colloidal stability and catalytic activity, which scale with surface coverage. Surface coverage of 90% MUA was found to be an optimal level of coverage where catalytic activity was observed, as well as the ability to recover and reuse for two catalytic cycles. The catalyst recovery by aggregation/re-dispersion and aqueous/organic phase transfer methods were achieved at pHs 4.1 and 8.7, respectively. The fundamental insight from this work allows for the understanding and designing the reusable colloidal metal nanoparticle catalysts with different surface functionalities and catalyzing the reaction at different pH conditions.

## Supplementary Materials

The following are available online at http://www.mdpi.com/2079-4991/8/5/339/s1. Figure S1: TEM images of AuNPs, Table S1: Hydrodynamic diameter and Zeta potential of AuNPs before and after MUA functionalization, Figure S2: UV-VIS spectra of MUA functionalization AuNPs with diameters of 5 nm, 13 nm, and 45 nm, Figure S3: UV-VIS absorption spectra of AuNP-MUA recorded at acidic and basic pH values. Figure S4: UV-VIS absorption spectra of AuNP-MUA recorded at different pH values varied from 9 to 3, Figure S5: Control experiment to show that ODA facilitates the phase transfer of AuNP-MUA, Figure S6: pH-triggered reversible phase transfer of AuNP-MUA studied using UV-VIS spectroscopy, Figure S7: The onset of phase transfer of AuNP-MUA from aqueous to CHCl3 phase, Figure S8: Time-resolved UV-VIS spectra for catalytic reaction of AuNPs as a function of MUA surface coverage, Figure S9: TGA curve for AuNP-MUA, Figure S10: Ability to recover the AuNPs functionalized with MUA different surface coverages, Figure S11: Fitting the absorbance data to pseudo-first-order reaction kinetics.
