# Supplementary material for: pH-Responsive Mercaptoundecanoic Acid Functionalized Gold Nanoparticles and Applications in Catalysis
Source: Nanomaterials (Basel). 2018 May 17;8(5):339. doi: 10.3390/nano8050339 (PMC5977353; doi:10.3390/nano8050339)
Supplement: Supplementary file 1 [file nanomaterials-08-00339-s001.pdf]

nanomaterials

Supplementary Information

pH-Responsive Mercaptoundecanoic Acid Functionalized Gold Nanoparticles and Applications in Catalysis

Contents: Page

S1. TEM images of AuNPs S2

S2. DLS of MUA functionalized AuNPs S3

S3. UV-VIS spectra of MUA functionalized AuNPs S3

S4. pH triggered reversible aggregation of AuNP-MUA S4

S5. The stability of the AuNP-MUA at different pHs S4

S6. Control experiment for phase transfer of AuNP-MUA S5

S7. pH triggered reversible phase transfer of AuNP-MUA S5

S8. Onset pH of phase transfer of AuNPs-MUA from aqueous to $CHCl_3$ layer S6

S9. Catalytic activity of AuNP-MUA as function of MUA packing density S6

S10. TGA curves of neat MUA and MUA functionalized AuNPs S7

S11. Recovery of AuNP functionalized with MUA different surface coverage S7

S12. Fitting the absorbance data to pseudo-first-order reaction kinetics S8

S1.TEM images of AuNPs.

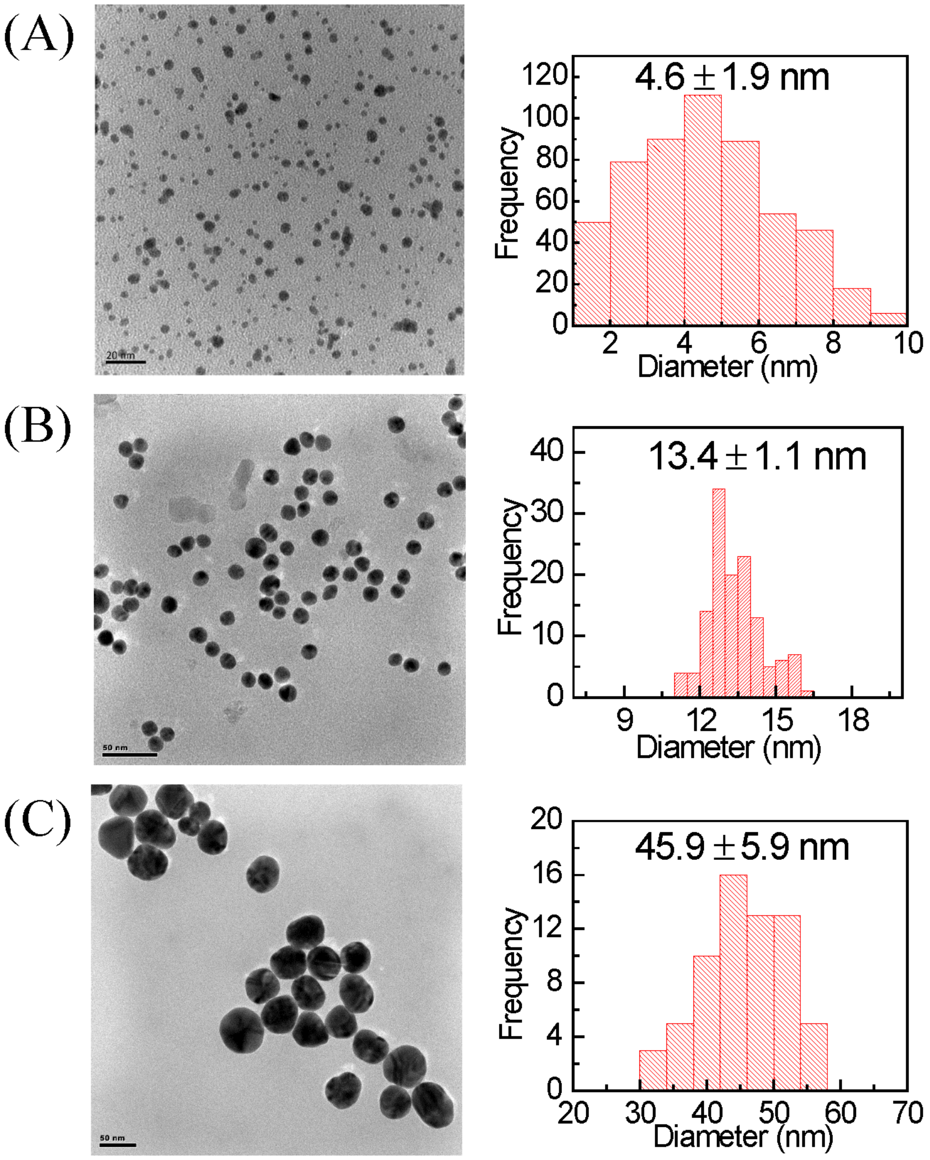

Figure S1. TEM images and histograms of distribution of sizes of AuNPs with diameter of (A) 5 nm, (B) 13 nm, and (C) 45 nm. The scale bars in the images (A), (B), and (C) are 20 nm, 50 nm, and 50 nm, respectively.

S2. DLS of MUA functionalized AuNPs.

Table S1. Hydrodynamic diameter and Zeta potential of AuNPs before and after MUA functionalization.

Nominal particle size /nm	Hydrodynamic diameter (nm)		Zeta potential (mV)	
	Before	After	Before	After
5	6.5 (±2.1)	11.1(±3.5)	-63.1(±1.9)	-42.6(±9.0)
13	16.3(±2.7)	18.3(±1.8)	-32.3(±1.0)	-39.8(±1.0)
45	48.4(±5.3)	49.2(±6.4)	-35.0(±1.8)	-41.6(±1.2)

S3. UV-VIS spectra of MUA functionalized AuNPs.

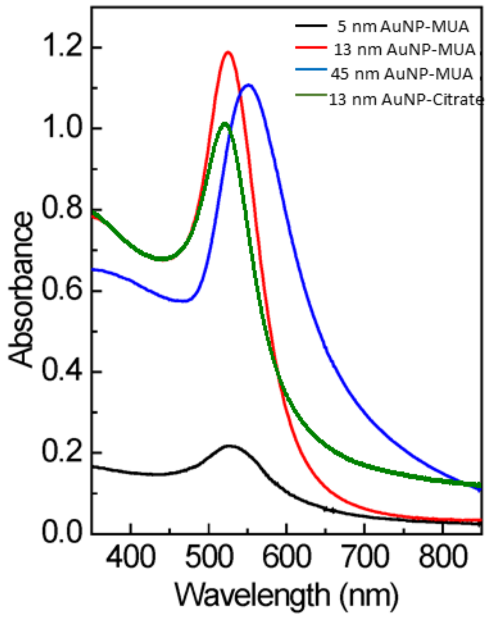

Figure S2. UV-VIS spectra of MUA functionalization AuNPs with diameters of 5 nm, 13 nm, and 45 nm. Green spectra represent 13 nm citrate-stabilized AuNP.

S4. pH-triggered reversible aggregation of AuNP-MUA.

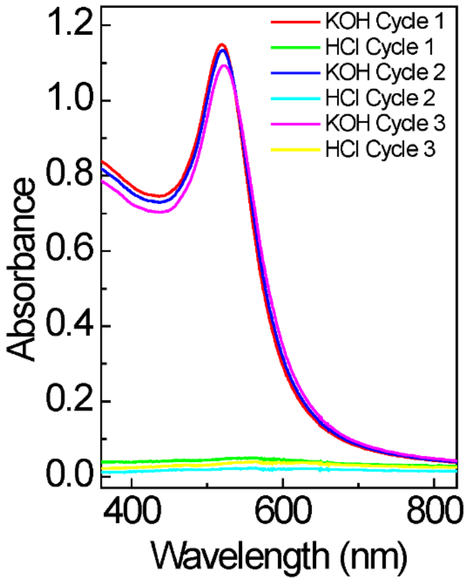

Figure S3. UV-VIS absorption spectra of AuNP-MUA recorded at acidic and basic pH values. The pH was varied by adding 10 μL of appropriate concentrations of HCl or KOH solutions. The process of aggregation/disaggregation of AuNP-MUA by changing pH was repeated up to three cycles.

S5. The stability of the AuNP-MUA at different pHs.

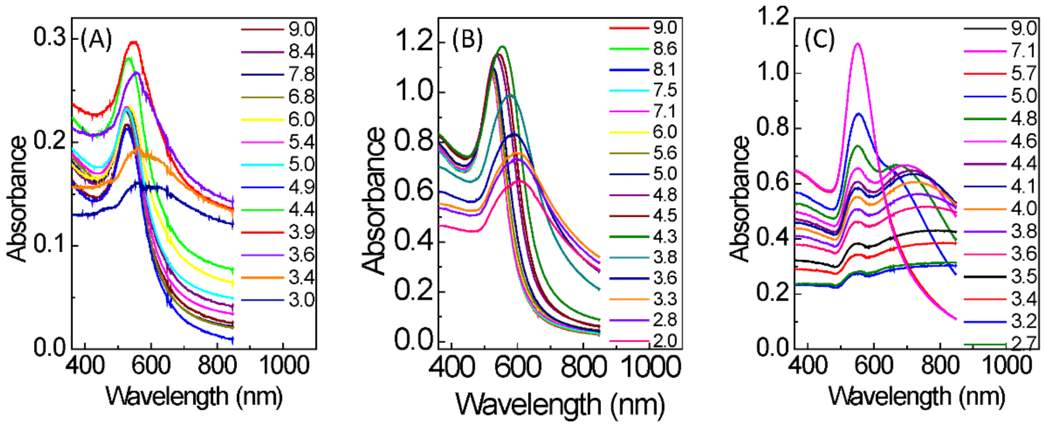

Figure S4. UV-VIS absorption spectra of AuNP-MUA recorded at different pH values varied from 9 to 3. (A), (B), and (C) are 5 nm, 13 nm, and 45 nm diameter AuNP functionalized with MUA, respectively.

S6. Control experiment to show that ODA facilitates the phase transfer of AuNP-MUA.

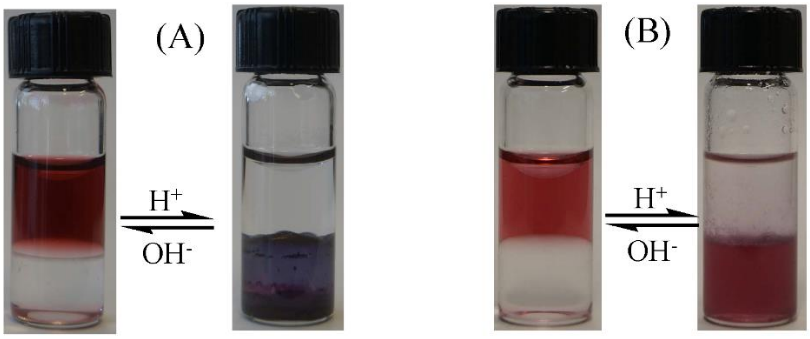

Figure S5. Control experiment to show that octadecylamine (ODA) facilitates the phase transfer of AuNP-MUA. (A) Photographs of glass vials containing aqueous solutions (top) of AuNP-MUA in contact with chloroform (bottom) without adding ODA. The initial aqueous solution of the AuNP-MUA in the vial was red in colour with pH > 4.1 (left). By adding 0.1 M HCl, the pH of the AuNPs aqueous solution was gradually decreased below 4.1 (right). After vigorous shaking and storing for about 2 min, the thin film of aggregated AuNPs, formed at water–chloroform and chloroform–glass wall interfaces. Re-dispersion of the AuNPs back to the aqueous phase is observed by adjusting the pH of the aqueous medium to > 4.1, by adding 0.1 M NaOH. (B) Photographs show the pH-triggered reversible phase transfer of AuNP-MUA between water and $CHCl_3$ layers with ODA, by switching the pH. The left side vial contains well-dispersed AuNP-MUA in an aqueous phase (top layer) at basic pH, and the right side vial contains AuNP-MUA transferred into the $CHCl_3$ phase (bottom layer), after adding HCl and vigorous shaking and storing for about 2 min.

S7. pH-triggered reversible phase transfer of AuNP-MUA studied by UV-VIS spectroscopy.

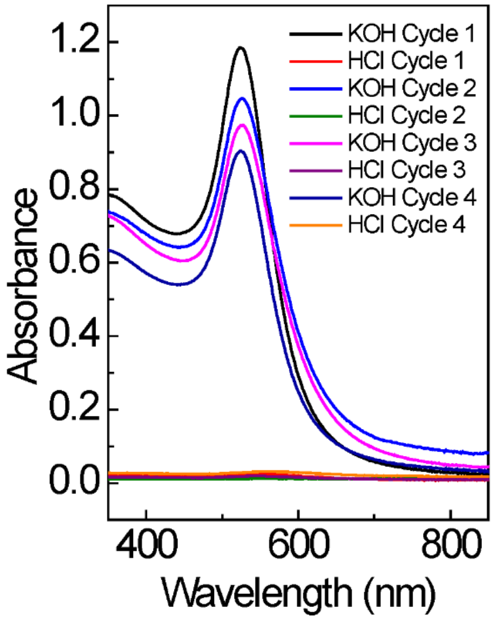

Figure S6. pH-triggered reversible phase transfer of AuNP-MUA studied using UV-VIS spectroscopy. UV-VIS absorption spectra of AuNP-MUA was recorded from theaqueous layer. The pH was varied by adding appropriate concentrations of HCl or KOH solutions. The direct phase transfer of AuNP-MUA between the aqueous and $CHCl_3$ phases was repeated for up to four cycles by changing the pH of the aqueous medium.

S8. Onset pH of phase transfer of AuNPs-MUA from aqueous to $CHCl_3$ layer.

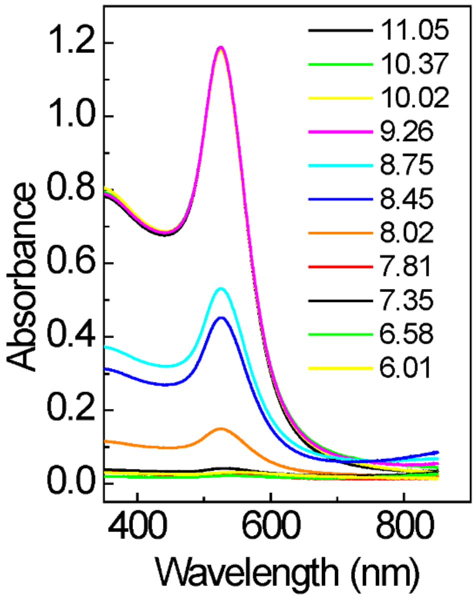

Figure S7. The onset of phase transfer of AuNP-MUA from aqueous to $CHCl_3$ phase was studied using the pH titration, and monitored the LSPR peak of nanoparticles in the aqueous phase from pH 11 to pH 6, by using UV-VIS spectroscopy.

S9. Catalytic activity of AuNP-MUA as a function of MUA packing density on AuNPs.

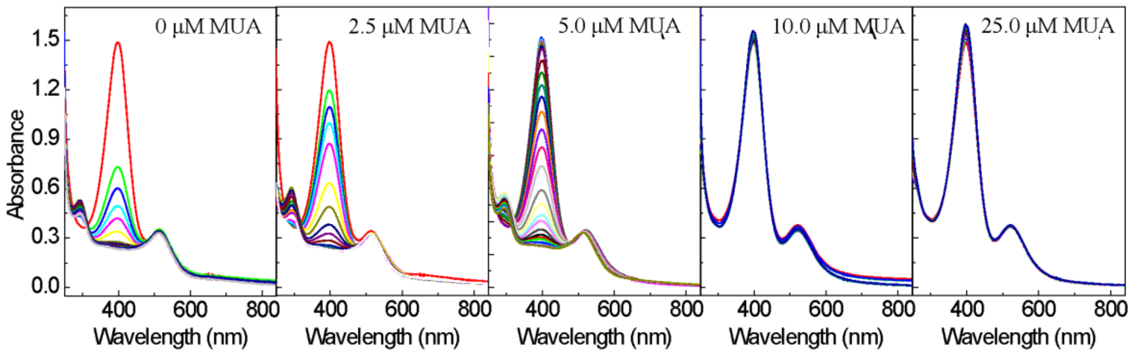

Figure S8. Time-resolved UV-VIS spectra of the 4-nitrophenol reduction reaction catalyzed by AuNP, functionalized with 0, 2.5, 5.0, 10.0, and 25.0 μM concentrations of MUA. Individual spectra were acquired every 0.2 mins. For visual clarity, spectra intervals shown here are at 0.2 min (0 and 2.5 μM), 1 min (5 μM), and 2 mins (10 and 25 μM).

S10. TGA curves of neat MUA and MUA functionalized AuNPs

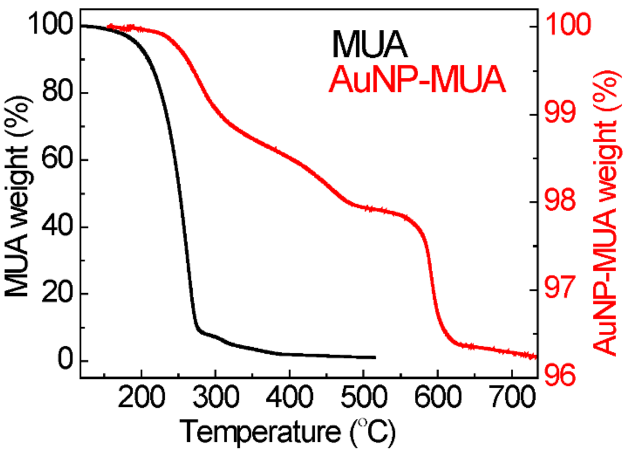

Figure S9. Thermogravimetric analysis (TGA) curves of neat MUA (black curve) and MUA functionalized AuNPs (red curve).

MUA surface coverage on 13.4 nm AuNPs was determined by TGA analysis. TEM provides an average core diameter of 13.4 nm for AuNPs. The NPs were modeled as spheres with radius (*r*) and assuming a size distribution with low polydispersity. Utilizing the weight fraction of MUA (w_l) (Figure S9), the weight fraction of the NP core (w_{NP}), mass of an individual particle (m_{NP}), surface area of an individual particle (SA_{NP}), molecular weight of ligand (MW_l), and Avogadro's Number (N_{AV}), the surface coverage per square nanometer (SC_{nm^2}) was calculated using the equation below.

$$SC_{nm^2} = \frac{m_{NP}.w_l.N_{AV}.}{w_{NP}.MW_l.SA_{NP}}$$

Under our experimental conditions, the weight fraction of MUA was 3.7 % (Figure S9). Since the average size of the NP is ~ 13.4 nm in diameter (Figure S1), the nominal packing density of MUA on the AuNPs was 4.56 molecules/nm^2.

S11. Recovery of AuNP functionalized with different MUA surface coverage

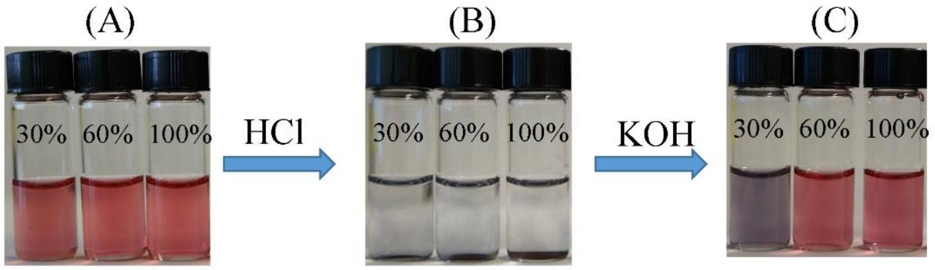

Figure S10. Ability to recover the AuNPs functionalized with MUA different surface coverages by using the aggregation/redispersion method. (A) The vials containing well-dispersed AuNP-MUA at basic pH. (B) The vials containing aggregated and settled AuNP-MUA at acidic pH. (C) The addition of KOH to aggregated AuNP-MUA, which only recovered AuNP with 60% and 100% MUA surface coverages.

S12. Fitting the absorbance data to pseudo-first-order reaction kinetics.

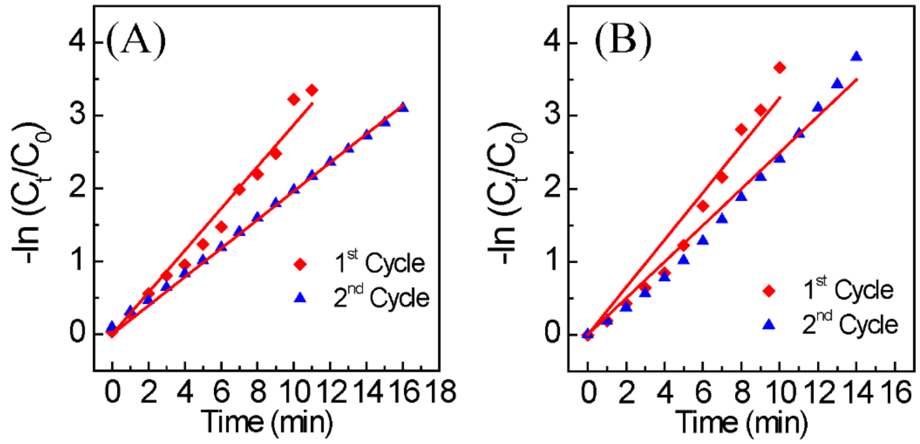

Figure S11. Fitting the absorbance data to pseudo-first-order reaction kinetics with respect to 4-NP for AuNP-MUA recovered by (A) pH-triggered aggregation/redispersion method and (B) pH-triggered phase transformation method.
